# The Influence of Osteon Orientation on Surface Topography Parameters after Machining of Cortical Bone Tissue

**DOI:** 10.3390/ma16124293

**Published:** 2023-06-09

**Authors:** Paweł Zawadzki, Rafał Talar, Karol Grochalski, Mikołaj Dąbrowski

**Affiliations:** 1 Faculty of Mechanical Engineering, Poznan University of Technology, Maria Sklodowska-Curie Square 5, 60-965 Poznan, Polandkarol.grochalski@put.poznan.pl (K.G.); 2 Adult Spine Orthopaedics Department, Poznan University of Medical Sciences, 61-545 Poznan, Poland; mdabrowski@ump.edu.pl

**Keywords:** orthogonal cutting, surface topography, cortical bone, abrasive machining, isotropy

## Abstract

Mechanical processing of cortical bone tissue is one of the most common surgical procedures. A critical issue accompanying this processing is the condition of the surface layer, which can stimulate tissue growth and serve as a drug carrier. A comparison of the surface condition before and after orthogonal and abrasive processing was conducted to validate the influence of bone tissue’s processing mechanism and orthotropic properties on the surface topography. A cutting tool with a defined geometry and a custom-made abrasive tool was used. The bone samples were cut in three directions, depending on the orientation of the osteons. The cutting forces, acoustic emission, and surface topography were measured. The level of isotropy and the topography of the grooves showed statistical differences relative to the anisotropy directions. After orthogonal processing, the surface topography parameter Ra was determined from 1.38 ± 0.17 μm to 2.82 ± 0.32. In the case of abrasive processing, no correlation was found between the orientation of osteons and topographical properties. The average groove density for abrasive machining was below 1004 ± 0.7, and for orthogonal, it was above 1156 ± 58. Due to the positive properties of the developed bone surface, it is advisable to cut in the transverse direction and parallel to the axis of the osteons.

## 1. Introduction

Orthopaedic surgeons frequently employ cortical bone tissue machining, a highly invasive technique [1]. It is primarily used for hip and knee arthroplasty, limb-fracture surgery, spine arthroplasty, and other joint-replacement procedures [2,3]. The procedure involves machining cartilage and bone tissue surfacing methods such as drilling, cutting, and milling [4]. However, these mechanical impacts emit thermal energy and can cause tissue damage, thereby increasing recovery time [5,6]. Despite the popularity of standardized surgical procedures, there have been no changes in processing technology or the availability of specialized surgical equipment.

Research on bone-tissue-cutting analysis mainly focuses on evaluating cutting forces, crack propagation, acoustic emission, thermodynamic phenomena, and chip morphology. The dominant modelling method for assessing the cutting process is orthogonal cutting. Liao et al. [7] and Bai et al. [8] extensively researched this field. Liao et al. [7] utilized a tool with a rake angle of *γ* = 8° and a clearance angle of *α* = 8°, which was also adopted by Liao et al. [9] for simulating bone milling procedures. Similarly, Bai et al. [8] employed a single tool with a rake angle of *γ* = 10° and a clearance angle of *α* = 7°. Jacobs et al. [10] conducted pioneering research using a more comprehensive range of tools with rake angles of *γ* = −5, 0, 15, 35, and 45°, although clearance angle information was not provided. Wiggins et al. [11] introduced a more comprehensive range of angles by using rake angles of *γ* = −10°, 10°, and 40° and a clearance angle of *α* = 10°. Although all studies showed the influence of the anisotropic bone structure on the results, the limited depth range of the cut and the cutting tool geometry used in these studies do not provide a comprehensive understanding of the entire process’s complexity.

An essential but still not fully understood issue is the topography of the surface of bone tissue and its influence on healing processes. Two main reasons for measuring surface topography are quality control and (perhaps most importantly) predicting surface performance [12]. Surface topography parameters are among the essential functional characteristics of materials [13]. The level of roughness is significant for the attachment of osteoblast cells and mineralization phenomena. Deligianni et al. [14] evaluated the influence of roughness on the response of human bone marrow cells. Cell adhesion, proliferation, and detachment force were sensitive to surface roughness and increased with its growth. Surface roughness generally improved the short- and long-term response of bone marrow cells in vitro. On the other hand, too much roughness, and thus numerous injuries to live cells, can limit the reconstruction of bone structures. Rumpler et al. [15] demonstrated a 2.8- to 7.3-times higher resorptive activity of osteoclasts on rough bone surfaces than on polished surfaces. These data strongly indicate that surface topography plays a crucial role in the resorptive activity of osteoclasts, including all previous stages, such as acquisition, attachment, and differentiation of precursor cells. Similar studies, however, were conducted on titanium implant surfaces by Tabassum et al. [16], indicating that a more significant amount of bone debris is located on rough surfaces, enhancing the osteogenic response. These bone fragments are presumed to act as miniature autografts, thus significantly strengthening osteogenesis around the implant. Ndaruhadi et al. [17], in their bone tissue turning studies, showed that surface roughness obtained does not affect cutting speed.

The importance lies in the tool material, its shape, and the direction of cutting relative to the orientation of osteons. The machining method has a significant impact on the surface topography of the material [18]. Similar results are indicated by Ni et al. [19], showing that for a feed rate of 30 mm/s, a surface roughness of 5.79 μm was obtained, and for 20 mm/s, it was 9.48 μm. Researchers also suggest that roughness increases by increasing the feed rate, decreases, and stabilizes. Surface roughness analysis using contact and non-contact methods of holes obtained with two drilling techniques, conventional and ultrasonically assisted, was carried out by Alam et al. [20]. The obtained *Ra* roughness values were similar in both cases and ranged from 1 < *Ra* < 1.5. The surface topography of the hole walls is crucial because it affects the strength of the connection between the screw and the implant, which is necessary for early and healthy bone growth [21].

In this paper, the authors measured cortical bone tissue’s topography subjected to orthogonal cutting and abrasive machining. During the experimental tests, cutting forces were recorded at a constant cutting speed and a controlled depth of cut. The surface topography was evaluated using a stylus profilometer. The main focus was on analysing the influence of the orientation of the bone structure on the concentration of grooves and the overall roughness of the surface. This study presents a comprehensive analysis of bone surface topography for the first time in light of its potential significance in designing treatment methods. The novelty of the research confirms the influence of the anisotropic properties of the bone tissue on the properties of the surface layer, which may be necessary during surgical treatment.

## 2. Materials and Methods

This research focused on investigating the technological effects of orthogonal cutting on cortical bone tissue using a cutting tool with a specific geometry. This study involved measuring cutting forces and examining surface roughness to determine the overall characteristics of the bone tissue. Orthogonal cutting, commonly used in basic research and industry, was chosen for this study. Before orthogonal cutting, a proprietary tool subjected the samples to abrasive machining. Cortical bone tissue, which forms the fundamental building block of the human skeletal system and is often operated on by orthopaedic surgeons, was used due to its 5–10% porosity [22] and its ability to carry a significant portion of the load on long bones [23,24,25,26]. Porosity also affects mechanical strength and elasticity [27]. A study found that variable porosity can cause stress concentration and initiate microcracks [28], but compression cracks can also increase compressive and tensile strength [29]. To ensure the stability and repeatability of the experimental studies, cortical bone tissue was selected for its homogeneous structure. The extensive studies allowed for a broad examination of bone tissue machining through cutting.

### 2.1. Bone Characteristics and Specimens

**Figure 1:**
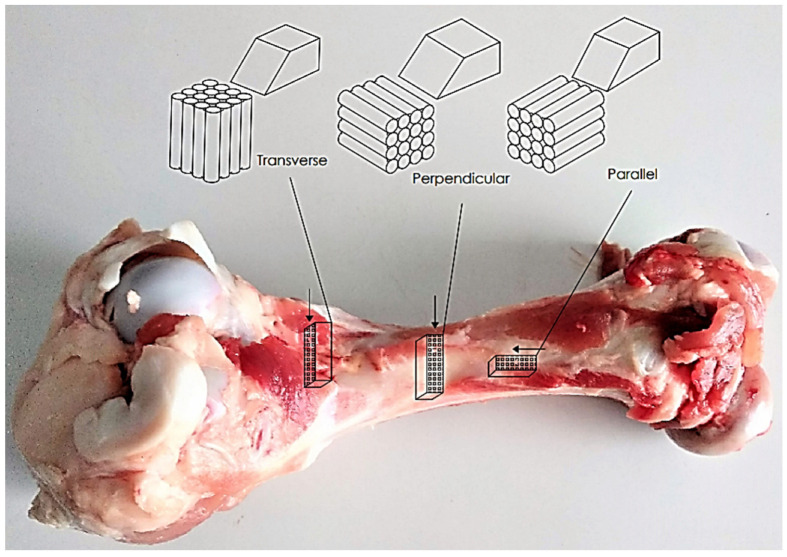
Preparation of bone specimens for transverse, perpendicular, and parallel directions of osteon orientation.

The composition of cortical tissue is a combination of various materials, including hydroxyapatite mineral [30], organic components, and water [31,32]. The XRF method has shown that bone tissue comprises calcium, phosphorus, potassium, and other elements in trace amounts [33]. The basic building blocks of cortical bone tissue are osteons, which are elliptical cylinders with a diameter of 100–300 µm and a length of 3–5 mm [34,35,36]. Osteons surround Haversian canals and are separated from the interstitial bone tissue by a thin layer of an amorphous substance called a cement line. Each osteon comprises concentric lamellae, where bone cells reside in ellipsoidal spaces [34,35]. The composition and properties of cortical bone tissue differ from those of cancellous bone tissue [37]. The elastic modulus in the longitudinal direction is higher than in the transverse direction. Studies suggest that bone tissue should be cut in three directions (see Figure 1). The interstitial matrix has a slightly higher elastic modulus than osteons [38,39]. The compressive strength of bone is around 100–130 MPa [40]. Healthy, mature cattle femurs were acquired from a local slaughterhouse and preserved in Ringer’s fluid. The bones were taken from the central shaft, and the literature suggests that bone mechanical properties are comparable to human cortical bone [41].

Samples were cut with a hand tool and then shaped using a Buehler Isomet 4000 sample cutting linear saw equipped with a cBN cutting wheel. Samples measuring 8 mm × 8 mm × 4 mm were collected from three different orientations due to the anisotropic nature of the cortical tissue [42]. Frozen bone samples can be utilized in research to investigate the mechanical properties of bone [43]. Moreover, the literature indicates no variation in mechanical properties between fresh and frozen cortical bone [44,45].

### 2.2. Cutting Tools Geometry

**Figure 2:**
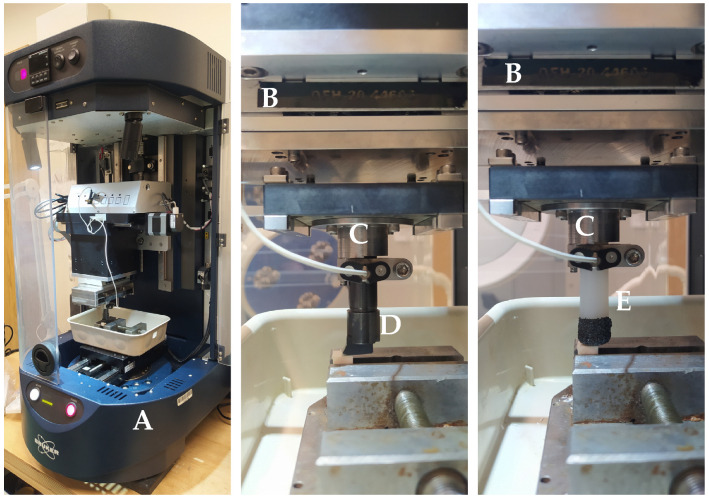
The measurement station: (**A**) Tribotester Bruker UMT, (**B**) force sensor DFM−20, (**C**) cutting tool holder, (**D**) orthogonal cutting tool, and (**E**) abrasive cutting tool.

This study used two types of tools: a cutting edge with a defined geometry (for orthogonal processing, see Figure 2D) and grinding wheels with undefined geometry (see Figure 2E). Avanti-Tools Sp produced the high-speed steel orthogonal cutting tool z o. o. in Poznań, Poland. This tool had a cutting edge width of 10 mm, rake angle *γ* = 30°, clearance angle *α* = 10°, and was fixed in a custom-made holder constructed of structural steel. The grinding tool consisted of a polyamide (PA6) shaft, at the end of which CBN grains were mounted—Cerabon CBN 10 Ti 50/60 (B 301) (CERATONIA GmbH & Co. KG, Ebelsbach, Germany) in an epoxy resin matrix with a hardener from SP-TEX Sp. z o.o. The shaft diameter was 12 mm, and the grain size ranged from 300 to 250 μm.

### 2.3. Experimental Setup

A measurement system was created to investigate cortical bone tissue’s grinding and orthogonal cutting procedure. The first step subjected six samples from each orientation to abrasive processing. Then, three samples were subjected to orthogonal cutting, and along with the remaining three, they were subjected to topography analysis.

**Figure 3:**
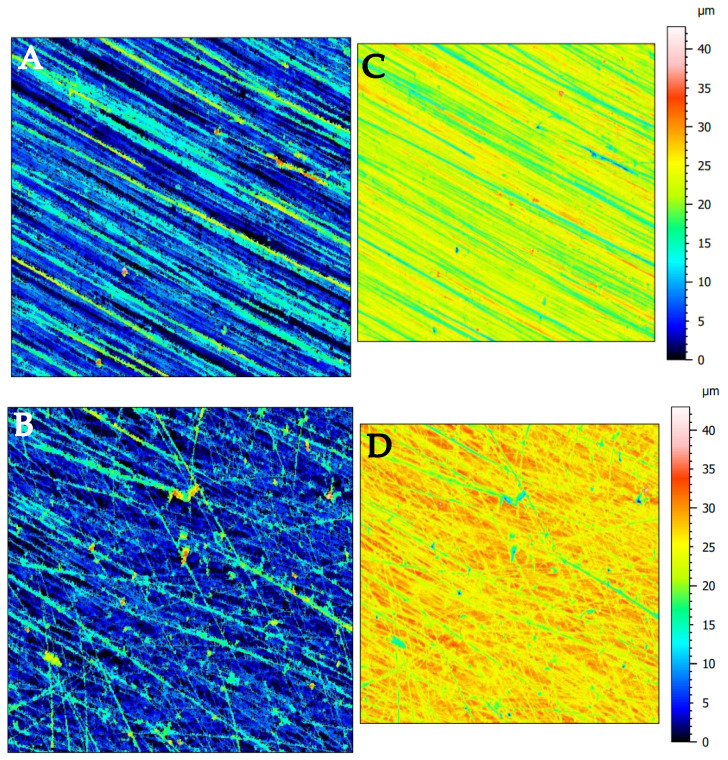
Surface topography of the sample before and after abrasion: visualization of grooves on the surface ((**A**)—sample, (**B**)—after machining) and overall visualization of the surface topography ((**C**)—sample, (**D**)—after machining).

The tool motion was carried out using UMT Bruker (Billerica, MA, USA) tribotester drives fitted with a 3-axis motion system and high-precision stepper motors (refer to Figure 3). The DFM-20 two-axis force sensor recorded forces within the 0.05 to 235 N range. The force sensor has a measuring resolution of 0.01 N, a non-linearity of 0.02%, and can sample data at 1000 Hz. This sensor allows for precise measurement of force and position in three dimensions.

The cutting depth of 25 μm was chosen based on previous experimental studies—this value provides the highest sensitivity to the influence of osteon orientation, minimizing disturbances caused by crack propagation [46]. To reduce the influence of thermal effects on the cutting process, a constant cutting speed of *v_c_* = 30 mm/min was maintained, which research has shown to prevent thermal necrosis at speeds below this value [11]. The tangential force *F_S_* and the contact force *F_N_* were measured during the experiment. However, the analysis focused on the force *F_C_* as it is a primary factor in forming cracks.

The topography analysis of the surface was conducted using the Hommel T8000 contact profilometer. Detailed analysis of the recorded cutting force signals, acoustic emission, and friction coefficient was conducted using dedicated CETR (version 1.138.264F03) software for tribotesters. The entire analysis was supplemented with a one-way ANOVA to demonstrate the significance and convergence of the results.

## 3. Results and Discussion

### 3.1. Grinding Tests

Cortical bone tissue subjected to abrasive processing in three directions of osteon orientation was analysed topographically. The structure of the sample before and after abrasive processing is shown in Figure 3. As a result of the abrasive processing, the sample surface lost topographical orientation due to the cutting disc’s rotational movement. The overall assessment of surface topography did not show a significant influence of osteon orientation on the surface condition after processing, although a slight deviation from the general trend was observed in the transverse direction (see Table 1). The limited cutting range resulting from the small size of abrasive grains and the process’s stochastic nature explains the osteon direction’s lack of significant influence. At the microprocessing level, the complex nature of cortical bone tissue does not have a potential influence, at least on surface topography. The cutting depth is too low for the orientation to affect the tissue deformation character. The top structure’s isotropy level for transverse, perpendicular, and parallel directions was 32.6 ± 7.3%, 23.1 ± 6.14%, and 15.9 ± 8.39%, respectively.

At the given cutting depth of 25 μm and an average groove depth of approximately 5 μm, the groove topography indicates a limited and stochastic cutting process. However, the orientation direction of osteons does not have a visible effect on the parameters.

Abrasive processing ensures a uniform structure of groove topography and overall surface topography of the tissue regardless of osteon orientation and processing conditions. When using this processing method, the operator does not need to adhere to the tool’s direction of movement guidelines.

### 3.2. Orthogonal Cutting

**Figure 4:**
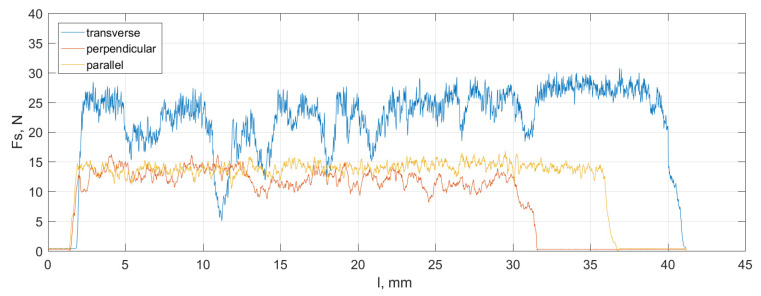
The cutting force values were recorded in the direction perpendicular to the tool motion during the orthogonal cutting of cortical bone samples in three cutting directions.

The overall values of the resultant cutting force *F_c_* take the lowest values in the perpendicular and parallel directions (see Figure 4). The most significant irregularity of the waveforms characterizes the transverse direction and the achievement of the highest *F_c_* values. Similar results were obtained by Bai et al. [8] and Liao and Axinte [7], indicating higher values of *F_c_* in the transverse direction. The increase in *F_c_* indicates an increase in shear resistance, and the intensification of fluctuations indicates an increase in crack propagation in chip formation. Due to the dominance of brittle crack propagation in bone tissue, a high value of *F_c_* indicates the accumulation of a significant amount of crack energy, which can lead to cracks penetrating the tissue structure. The regularity of *F_c_* profiles for perpendicular and parallel orientations suggests greater stability in chip formation processes, potentially affecting surface topography.

The crack propagation mechanism affects the topography of the bone tissue surface after processing. A noticeable correlation between *F_c_* and the *Spk*/*Sk* parameter based on the height distribution allows for a practical assessment of the cutting condition. As the wear progresses, the upper portion of the material ratio curve shifts downwards while the lower portion shifts upwards. In this case, the transverse direction had the lowest *Spk*/*Sk* value of 0.68, while the perpendicular and parallel directions had 0.95 and 0.92, respectively. This result indicates a higher surface complexity generated during cutting in the transverse direction.

**Figure 5:**
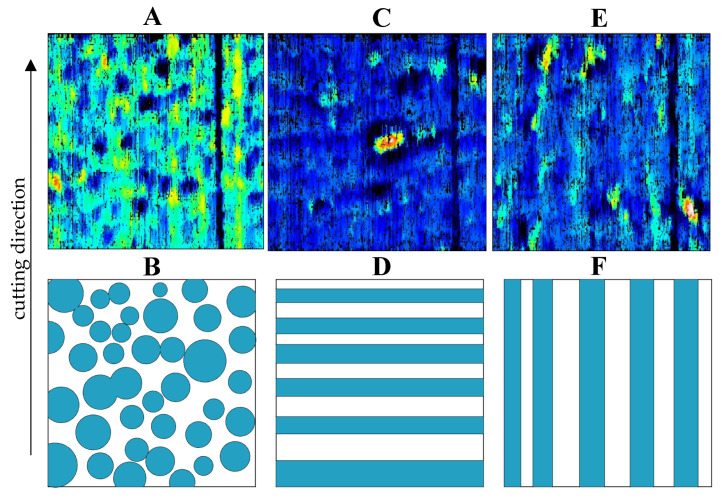
The surface topography of grooves after orthogonal cutting and visualization of the orientation of osteons in directions: (**A**,**B**) transverse, (**C**,**D**) perpendicular, and (**E**,**F**) parallel.

The topography of groove surfaces indicates high variability depending on the orientation of the osteons (see Figure 5B,D,F). The parameters *Vmc* and *Vvc* are insignificant, but *Vmp* and *Vvv* allow distinguishing between orientation directions. For transverse, perpendicular, and parallel directions, *Vmp* values of 0.21 ± 0.03, 0.38 ± 0.06, and 0.45 ± 0.02 mL/m^2^ were obtained, with *p* = 0.0049, and *Vvv* values of 0.31 ± 0.03, 0.71 ± 0.21, and 1.03 ± 0.22 mL/m^2^ were obtained, with *p* = 0.019. The statistical analysis of pairs presents a transitional character of the perpendicular direction. Figure 1D shows a potential arrangement of osteons in the tested samples. Figure 5A,B, transverse, corresponds to a point distribution of osteons, their cross-section, while Figure 5E,F, parallel, corresponds to a linear longitudinal cross-section distribution. The perpendicular direction does not show a clear pattern due to the non-uniform distribution of osteons (see Figure 5C,D).

The variability of surface topography depending on the cutting direction results from the composite nature of cortical bone tissue. According to recent studies, the Young’s modulus of the osteon, interstitial matrix, and cement line is 20, 21.87, and 7.4 GPa, respectively [47]. These differences affect the nature of crack propagation, as confirmed by studies such as those by Bai et al. [8] and Zawadzki and Talar [46].

**Figure 6:**
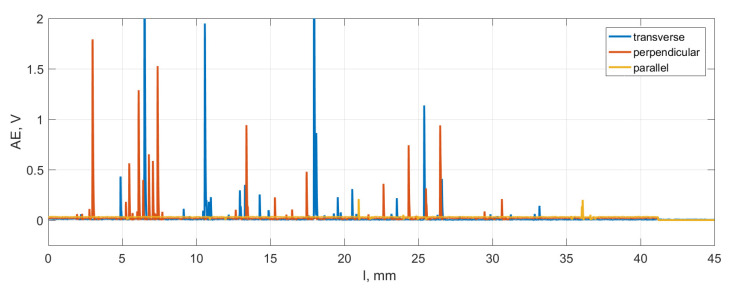
Acoustic emission signal (AE) was recorded during orthogonal cutting in three directions of osteon orientation.

The acoustic emission signal providing information about the occurrence of cracks is presented in Figure 6. The weakest cracks were recorded for the parallel direction due to the free propagation of cracks along the cement line. The highest frequency of cracks occurs in the perpendicular direction due to the irregular arrangement of composite structures. The strongest but less frequent cracks are observed for the transverse direction. According to the observations of Zawadzki and Talar [46], the freedom of delamination of osteons for cutting in the parallel direction may cause an increased depth of cutting when the material cracks along the cement lines without significant resistance.

**Figure 7:**
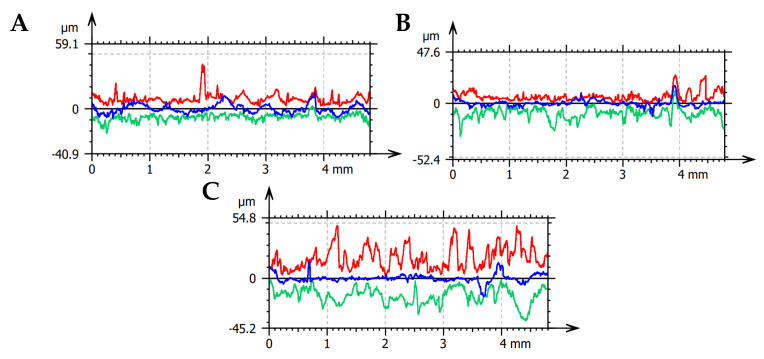
Roughness profiles in three directions of osteon orientation: (**A**) transverse, (**B**) perpendicular, and (**C**) parallel—maximum elevations (red color), maximum depressions (green color), average roughness profile (blue color).

Comparing the parameters of the 2D roughness profiles (see Figure 7), one can notice the similarity with crack propagation (see Figure 6). The registered maximum peak height of the profile, for transverse, perpendicular, and parallel directions, respectively, was 10.79 ± 1.86 μm, 16.77 ± 1.1 μm, and 20.27 ± 0.37 μm. In comparison, the maximum valley depth of the profile was 8.78 ± 0.96 μm, 16.87 ± 2.62 μm, and 38.65 ± 7.15 μm, respectively. Increased crack frequency resulting from transverse and perpendicular orientation reduces surface irregularities after machining (due to the shortened length of the crack). Interestingly, despite the accumulation of significant energy during machining (see Figure 1) in the transverse direction, the discontinuity of the osteon has a more negligible impact on surface topography than the crack propagation susceptibility. It is due to the search for potentially the shortest crack, albeit requiring the lowest amount of energy accumulation. Therefore, in the case of the transverse direction, cracks pass through the osteons, and in the parallel direction, they propagate freely along the cement line. For the perpendicular direction, the crack’s character is mixed. The arithmetic means deviation of the roughness profile was 1.38 ± 0.17 μm, 1.95 ± 0.34 μm, and 2.82 ± 0.32 μm for transverse, perpendicular, and parallel directions, respectively, achieving a level of finishing by machining.

The results presented in Table 1 show that the maximum and average depth of grooves increase from transverse through perpendicular to parallel, although the average density decreases. The p-value corresponding to the F-statistic of one-way ANOVA is lower than 0.05, suggesting that one or more treatments were are significantly different (see Table 2). Additionally, the Bonferroni and Holm tests indicate in detail that the pair of transverse and parallel is statistically distinguishable, consistent with the widely accepted transversely isotropic model of bone tissue. The perpendicular direction is a transitional phase between the transverse and parallel orientations. The parameter *Svk* confirms the analysis of groove depth, indicating the mean height of the protruding dales beneath the core at the level of 2.86 ± 0.55 μm for the transverse direction, 8.31 ± 2.58 μm for the perpendicular, and 13.15 ± 3.42 μm for parallel.

The recorded average width of elements in the unfiltered *Psm* profile ranges from 223 to 331 μm, while the osteon diameter is 150 to 300 μm. This may indicate the formation of potential traces after removing osteons or interstitial matrix fragments.

**Figure 8:**
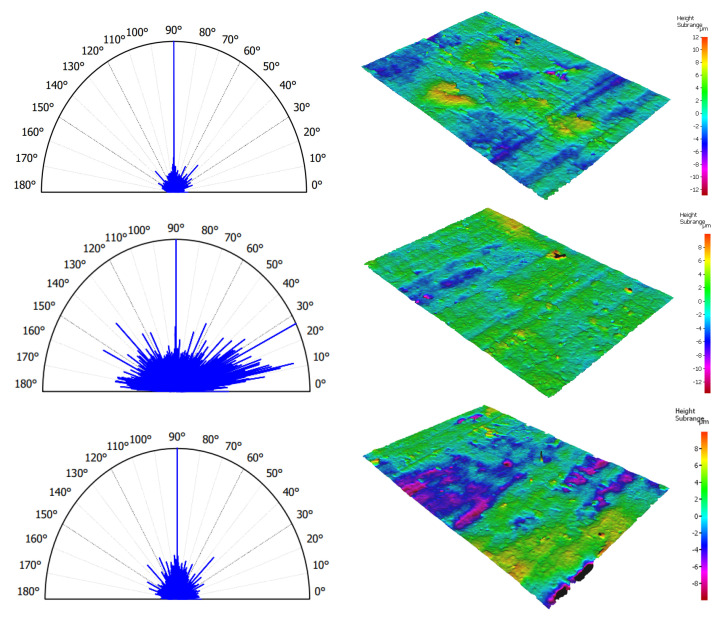
Level of isotropy and visualization of roughness for surfaces obtained with orthogonal cutting in three directions (from top): transverse, perpendicular, and parallel.

The varying level of surface isotropy correlates with the direction of cutting (see Figure 8). The following values of the isotropy coefficient were recorded for the transverse, perpendicular, and parallel directions: 6.25 ± 0.59, 73 ± 13, and 33.1 ± 9.46%, respectively. The *p* = 0.0064, less than 0.05, suggesting that one or more treatments differ significantly. Therefore, isotropy is distinguishable concerning the direction of cutting, and pairs of transverse–perpendicular and perpendicular–parallel are statistically distinguishable.

According to the research conducted by Schwartz et al. [48], a more developed surface topography positively impacts osteoblastic cell proliferation, differentiation, and matrix production in vitro. Surface topography is crucial as it affects the screw–implant connection strength, essential for early and healthy bone growth [21]. Therefore, cutting in a parallel direction is the best solution for forming the most developed surface layer. A comparative analysis of the surface topography results from Figure 3 with the results of Liao et al. [7] indicates the presence of similar surface-damage structures. In the transverse direction, distinct circular structures are observed, and fragments of osteons cut transversely to the axis. In the case of the parallel direction, longitudinal traces are visible, representing exposed cement lines due to damaged osteons. The arithmetic means deviation of the roughness profile was 1.38 ± 0.17 μm, 1.95 ± 0.34 μm, and 2.82 ± 0.32 μm for transverse, perpendicular, and parallel directions, respectively, achieving a level of finishing by machining.

### 3.3. Tribological Properties

From a tribological point of view, forming the surface layer is essential, primarily influenced by elastic–plastic deformations and the character of crack propagation in the tissue. Material decohesion during chip formation, caused by energy accumulation, is strongly erosive. The complex process of chip formation, composed of a series of microcracks along the cement lines, affects the surface topography. The friction coefficient recorded directly during orthogonal processing in the chip–tool contact zone was for transverse, perpendicular, and parallel, respectively: 0.86 ± 0.06, 0.79 ± 0.03, and 0.71 ± 0.08. The coefficient of friction (*COF*) is directly correlated with the cutting force, which results from the orientation of the osteons. The changes in cutting force values shown in Figure 4 indicate that the freest propagation of cracks occurs in the case of parallel direction, as evidenced by the *COF* value. The distinct depressions in Figure 8 result from uncontrolled decohesion along the horizontal cement lines. The distinct elevations for the transverse direction topography may indicate local deformations caused by the high cutting energy of osteons, whose elastic modulus is higher than the cement line. Elastic–plastic properties with local brittle behaviours characterize the cutting process.

## 4. Conclusions

This study performed experiments on the surface topography of cortical bone tissue after abrasion machining and orthogonal cutting. The effects of cortical bone anisotropy, machining method, cutting force, and coefficient of friction (*COF*) were also analysed.

The crack propagation mechanism affects the topography of bone tissue surface after machining. There is a correlation between *F_c_* and the *Spk*/*Sk* parameter based on the height distribution, allowing for a practical assessment of the cutting condition. The topography of groove surfaces indicates high variability depending on the orientation of the osteons. Parameters *Vmp* and *Vvv* allow for distinguishing between orientation directions, with statistical analysis showing a transitional character of the perpendicular direction. The variability of surface topography depending on the cutting direction results from the composite nature of cortical bone tissue. Differences in the Young’s modulus of the osteon, interstitial matrix, and cement line affect the nature of crack propagation. The acoustic emission signal provides information about the occurrence of cracks. The weakest cracks were recorded for the parallel direction, while the highest frequency of cracks occurs for the perpendicular direction, and the strongest but less frequent cracks are observed for the transverse direction. The maximum and average depth of grooves increases from transverse to parallel while the average density decreases. The perpendicular direction is a transitional phase between the transverse and parallel orientations. The parameter *Svk* confirms the analysis of groove depth, indicating the mean height of the protruding dales beneath the core.

However, the issue’s complexity level requires further, more complex research. Understanding the impact of the machining process on the surface topography and its dependence on the orientation of osteons is a severe issue for healing processes. Perhaps a specific surface topography, e.g., rich in numerous grooves and pores, will enable the placement of drugs or better adhesion of implants. This issue requires further analysis, and the above is an introduction.

## Figures and Tables

**Table 1:** Parameters of the grooves’ topography after grinding cortical bone tissue in three cutting directions.

Direction	Max. Depth of Grooves	Avg. Depth of Grooves	Avg. Groove Density	*Vmp*	*Vvv*
	µm	µm	cm/cm^2^	mL/m^2^	mL/m^2^
Transverse	74.2 ± 49	4.95 ± 0.62	957 ± 8	0.17 ± 0.06	0.59 ± 0.18
Perpendicular	30 ± 3.3	4.78 ± 0.04	988 ± 106	0.11 ± 0.01	0.49 ± 0.04
Parallel	24.7 ± 1.9	4.12 ± 0.08	1004 ± 0.7	0.11 ± 0.01	0.38 ± 0.01

**Table 2:** Parameters of the topography of the grooves after the orthogonal cutting of cortical bone tissue in three cutting directions.

Direction	Max. Depth of Grooves	Avg. Depth of Grooves	Avg. Groove Density
	µm	µm	cm/cm^2^
Transverse	16.6 ± 2.94	5.02 ± 0.84	1311 ± 42
Perpendicular	41.03 ± 13.89	6.86 ± 2.06	1246 ± 29
Parallel	66.59 ± 14.88	9.76 ± 1.32	1156 ± 58
*p*	0.0138	0.0403	0.0369

## Data Availability

Not applicable.
